# Heat inactivation of the Middle East respiratory syndrome coronavirus

**DOI:** 10.1111/irv.12261

**Published:** 2014-06-24

**Authors:** India Leclercq, Christophe Batéjat, Ana M Burguière, Jean-Claude Manuguerra

**Affiliations:** a Laboratory for Urgent Response to Biological Threats (CIBU), Environment and Infectious Risks Unit, Institut PasteurParis, France; b Université Paris Diderot, Sorbonne Paris Cité (Cellule Pasteur)Paris, France

**Keywords:** Heat, inactivation, MERS-CoV

## Abstract

The culture supernatants of the emerging Middle East respiratory syndrome coronavirus (MERS-CoV) were submitted to three temperatures over time and tested for infectivity by TCID_50_ method on Vero E6 cells. At 56°C, almost 25 minutes were necessary to reduce the initial titre by 4 log_10_. Increasing temperature to 65°C had a strong negative effect on viral infectivity as virucidy dropped significantly to 1 minute. On the contrary, no significant decrease in titre was observed after 2 hours at 25°C. These data might be useful in establishing biosafety measures in laboratories against MERS-CoV.

## Short report

The emerging Middle East respiratory syndrome coronavirus (MERS-CoV) was found to cause sporadic cases of severe acute respiratory infection. Between April 2012 and 26 April 2014, a total of 261 laboratory-confirmed cases of infection with MERS-CoV were reported, including 93 deaths, in nine different countries.^1^ To date, the viral transmission route is still not elucidated, although recent studies showed that tomb bats and camels may play a role as reservoirs or intermediate hosts.^2^,^3^ Coronaviruses are enveloped viruses, usually known to be fragile in the environment. However, enveloped viruses can persist in the environment for extended periods of time, even at 35°C.^4^ Understanding the potential effect of heat inactivation of the novel coronavirus is of significant value to elaborate proper public intervention measures.

A human strain of MERS-CoV was isolated in our laboratory from a French patient hospitalised in June 2013 after a nosocomial transmission.^5^ The viral strain MERS-CoV Hu/France–FRA2_130569/2013 (FRA2) was grown on MRC5 cells (RD-Biotech REF-84002) for the first passage and on Vero E6 cells (ATCC® CRL-1586) for the second passage. The cells were maintained in Dulbecco's modified Eagle's medium (DMEM 1X, GIBCO; Invitrogen, Saint Aubin, France) and supplemented with 5% foetal calf serum (FCS), antibiotics (0·1 units penicillin, 0·1 mg streptomycin per ml, GIBCO; Invitrogen) at 37°C in humidified 5% CO2 incubator. The cell culture supernatant was used for inactivation assays and whole-genome sequencing (manuscript under submission). Culture supernatants (500 μl) with a titre of 10^5·59^ TCID_50_ per ml were submitted to three temperatures over time and tested for infectivity by TCID_50_ method on Vero E6 cells as described previously, except that examination for cytopathic effect was performed after 6 days.^4^ Several time points were chosen (0, 0·5, 15, 30, 60 and 120 minutes). Each condition was performed in triplicates, and the whole experiment was accomplished twice. Experimental data from one experiment are shown in Table1. For each condition, we determined the virucidal activity of heat at 56 and 65°C, which corresponded to a reduction of 4 log_10_ of the titre according to the European Standards (NF EN 14476 available at http://www.afnor.org/; Table2). At 56°C, which is the common temperature used for inactivation of enveloped viruses, such as influenza viruses, and serum decomplementation, almost 25 minutes were necessary to reduce the initial titre by 4 log_10_. Increasing temperature to 65°C had a strong negative effect on viral infectivity as virucidy dropped significantly to 1 minute. Fifteen minutes at 65°C is more than sufficient to totally inactivate the sample. By contrast, no decrease in titre was observed after 2 hours at 25°C.

**Table 1:** TCID_50_ per ml values obtained at 56 and 65°C

	56°C					65°C				
		
Time (minute)	0·5	15	30	60	120	0·5	15	30	60	120
Sample 1	10^5·5^	10^0·67^	ND	ND	ND	10^4·67^	ND	ND	ND	ND
Sample 2	10^6·17^	10^1·0^	10^0·67^	ND	ND	10^2·00^	ND	ND	ND	ND
Sample 3	10^4·67^	ND	10^1·33^	ND	10^0·67^	10^3·67^	ND	ND	ND	ND

Time zero values were 10^5·59^ TCID_50_ per ml for each sample. ND: not detected (below the limit of virus detection which corresponded to 10^0·67^ TCID_50_ per ml). The whole experiment was performed twice with similar results (data not shown).

**Table 2:** Persistence times estimated using linear regression model for heat inactivation at 25, 56 and 65°C

Strain	Temperature	Slope (a)	*y*-Intercept (b)	*x*-Intercept (minute)	Virucidy (minute)
FRA2	25°C	0·006	5·25	NA	NA
	56°C	−0·16	5·09	30·7 [26·0; 35·2]	24·1
	65°C	−3·62	5·25	1·45 [1·0; 1·9]	1·1

Slope (a), *y*-intercept (b) and *x*-intercept values of the linear regression straight lines calculated from TCID_50_ values (ND values in Table1 were not included in the calculation). Virucidy corresponded to the duration necessary to obtain a fourfold reduction of the titre in log_10_. The values in brackets corresponded to the 99% confidence intervals. NA, not applicable. Each condition was performed in triplicates. The whole experiment was performed twice with similar results (data not shown).

Serum heat inactivation at 56°C for 30 minutes is a standard procedure in diagnostic laboratories to eliminate the potential complement interference in serological assays. Our results showed that this procedure is sufficient for viral inactivation as virus titres in blood are expected to be weaker than at the point of infection. This would be also sufficient for inactivation of viruses present in lower respiratory specimens, which are now recommended by WHO rather than nasopharyngeal swabs for viral diagnosis. For example, the FRA2 original clinical specimen (induced sputum) contained 6·5 × 10^7^ genome copies per ml for Orf1a calculated from the *C*_*t*_ values using a standard (manuscript under submission). These data might be also useful in establishing biosafety measures in laboratories against MERS-CoV.
